# Resection of unresectable hepatocellular carcinoma after conversion therapy with apatinib and camrelizumab: a case report and literature review

**DOI:** 10.3389/fonc.2024.1280805

**Published:** 2024-03-27

**Authors:** Xin-Liang Liu, Xiang-Ze Li, Yi-Fu Chu, Feng Liu, Hu Tian

**Affiliations:** ^1^ Shandong Provincial Qianfoshan Hospital, Shandong University, Jinan, China; ^2^ Department of Gastrointestinal Surgery, Shandong Provincial Third Hospital, Shandong University, Jinan, China; ^3^ Department of General Surgery, The First Affiliated Hospital of Shandong First Medical University & Shandong Provincial Qianfoshan Hospital, Jinan, China

**Keywords:** hepatocellular carcinoma, conversion therapy, apatinib, camrelizumab, tyrosine kinase inhibitors, immune checkpoint inhibitors, surgical resection

## Abstract

Hepatocellular carcinoma is a rather common malignant tumor. Most patients with hepatocellular carcinoma receive their diagnosis at an advanced stage, at which surgical resection is no longer appropriate. A growing body of research has demonstrated the value of convention therapy for patients with intermediate-stage hepatocellular carcinoma, while specific application protocols and treatment guidelines are not well developed. Emerging clinical researches suggest that a tyrosine kinase inhibitor in combination with an immune checkpoint inhibitor is a reasonable strategy for unresectable hepatocellular carcinoma. However, there are relatively few reports on the efficacy of apatinib and camrelizumab in the treatment of hepatocellular carcinoma. We were able to successfully remove one patient’s hepatocellular carcinoma after 8 cycles of conversion therapy with apatinib (250 mg orally every day) and camrelizumab (200 mg intravenously every 2 weeks). The patient continued to receive the same dose of 16 cycles of apatinib and camrelizumab after hepatectomy. By the time of this study, the patient has completed 18 months of follow-up, and no tumor recurrence or metastasis was found in tumor markers and imaging examinations. Apatinib in combination with camrelizumab is an effective therapy for the treatment of advanced hepatocellular carcinoma, and surgical resection after this conversion therapy may provide patients with long-term oncological benefits. However, this requires more samples to validate the conclusion.

## 1 Introduction

One of the most prevalent malignant tumors in the world is primary liver cancer (1, 2), of which the most prevalent type is hepatocellular carcinoma (HCC) (3). With a 5-year survival rate of more than 70%, surgery is the most efficient curative therapy for HCC in its early stage (4–7). Unfortunately, the majority of patients have advanced disease when they first appear (8, 9), making them unsuitable for surgery and giving them a terrible prognosis with a 5-year survival rate of only 10%–20% (10). A significant medical need is the creation of novel medicines for the efficient management of advanced HCC. As a result, the idea of conversion therapy—which refers to turning an HCC that cannot be surgically removed into one that can—was created (11). Conversion therapy has a number of options, including systemic and localized treatments. On the one hand, systemic therapy can be used alone to treat HCC, and the most popular form of treatment is the admixture of two medications: tyrosine kinase inhibitors (TKIs) and immune checkpoint inhibitors (ICIs). On the other hand, local therapy usually refers to the treatment of hepatocellular carcinoma using radiation therapy, transarterial chemoembolization (TACE), hepatic arterial infusion chemotherapy (HAIC), and selective internal radiation therapy (SIRT), and it is often used in conjunction with one or both of the TKIs and ICIs. Although conversion therapy is of great interest in the treatment of HCC, its application and treatment guidelines are not well developed. As we know, apatinib is a highly selective small-molecule tyrosine kinase inhibitor of vascular endothelial growth factor receptor 2 (VEGFR-2). And results from phase II clinical trials have shown that apatinib as a first-line treatment has potential survival benefits for Chinese patients with advanced HCC (12). Camrelizumab is a humanized PD-1 monoclonal antibody that has a high affinity with PD-1, high occupancy of circulating T-lymphocyte receptors (85% at a dose of 200 mg) (13), and a good tolerance and curative effect on a variety of solid tumors, including HCC (14). Currently, more and more studies have proved the clinical value of TKIs plus ICIs in patients with unresectable HCC, while apatinib combined with camrelizumab in patients with advanced HCC, only a few studies report this issue. Thus, we reported a case that apatinib and camrelizumab were used to treat a patient with unresectable HCC. The results show that conversion therapy with apatinib and camrelizumab is a safe and efficient method for resecting originally unresectable HCC, which may improve the patient’s long-term survival.

## 2 Case presentation

**Figure 1:**
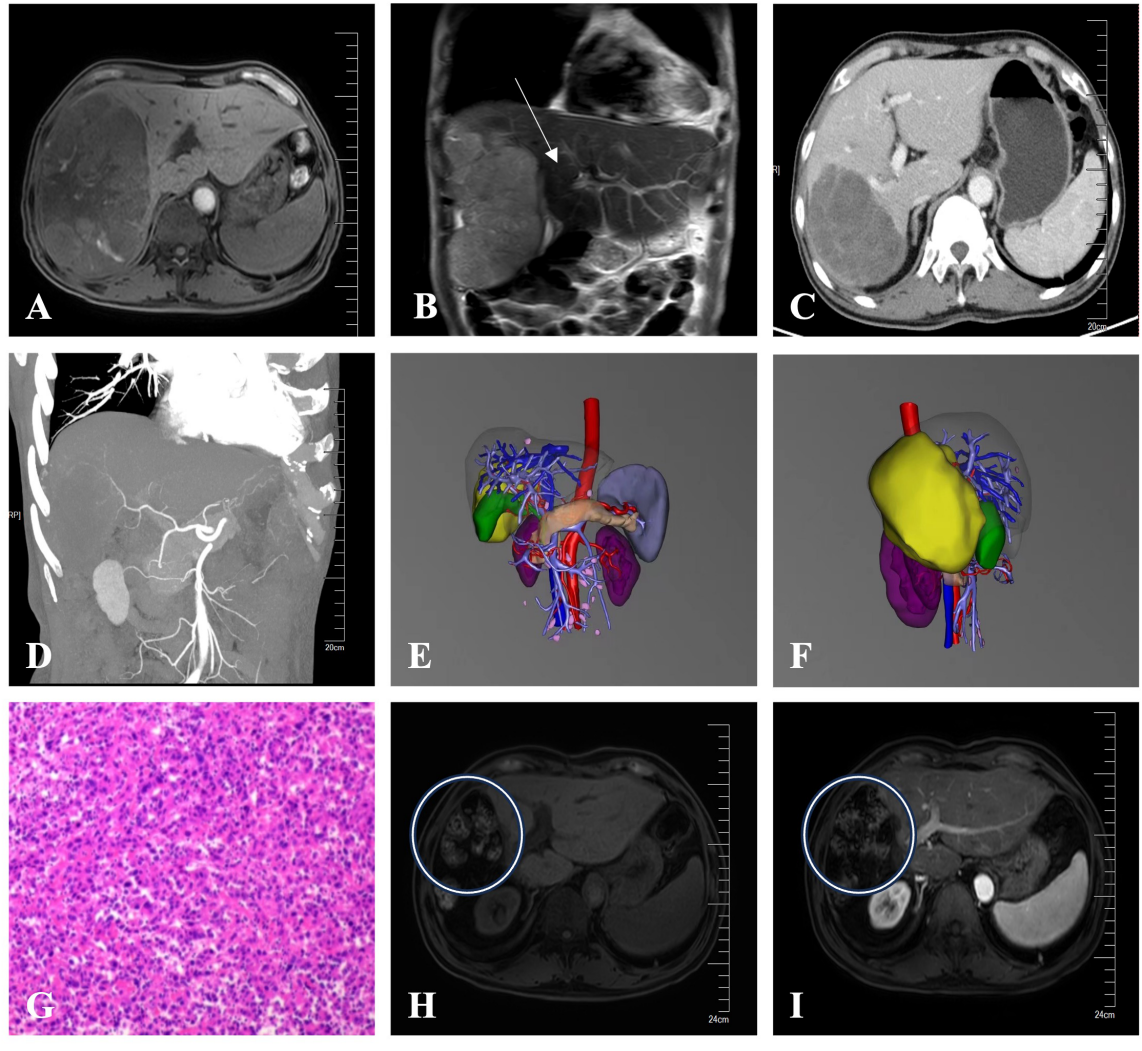
**(A)** Before conversion therapy, a dynamic contrast-enhanced abdominal MRI showed a tumor in the right lobe of the liver that was roughly 19.1x14.6x10.4cm in size. **(B)** Before conversion therapy, an abdominal coronal MRI showed the distal end of the right branch of the portal vein are poorly visualized, which is consistent with invasion by HCC (white arrow). **(C)** The tumor in the right lobe of the liver shrank to 8.6x6.9cm in size after conversion therapy, according to an abdominal contrast-enhanced CT scan. **(D)** CTA revealed roughly normal blood flow across the hepatic arteries. **(E, F)** The liver’s three-dimensional reconstruction from the positive and right-side views showed the location of the tumor (the yellow part). **(G)** The target lesions had coagulative necrosis, hyperplasia of the surrounding fibrous tissue, and lymphocyte infiltration, according to the postoperative pathology (HE staining). **(H, I)** The patient’s latest dynamic contrast-enhanced abdominal MRI revealed encapsulated fluid (white circles) with no discernible enhancement at the site of liver operation after the hepatectomy.

**Figure 2:**
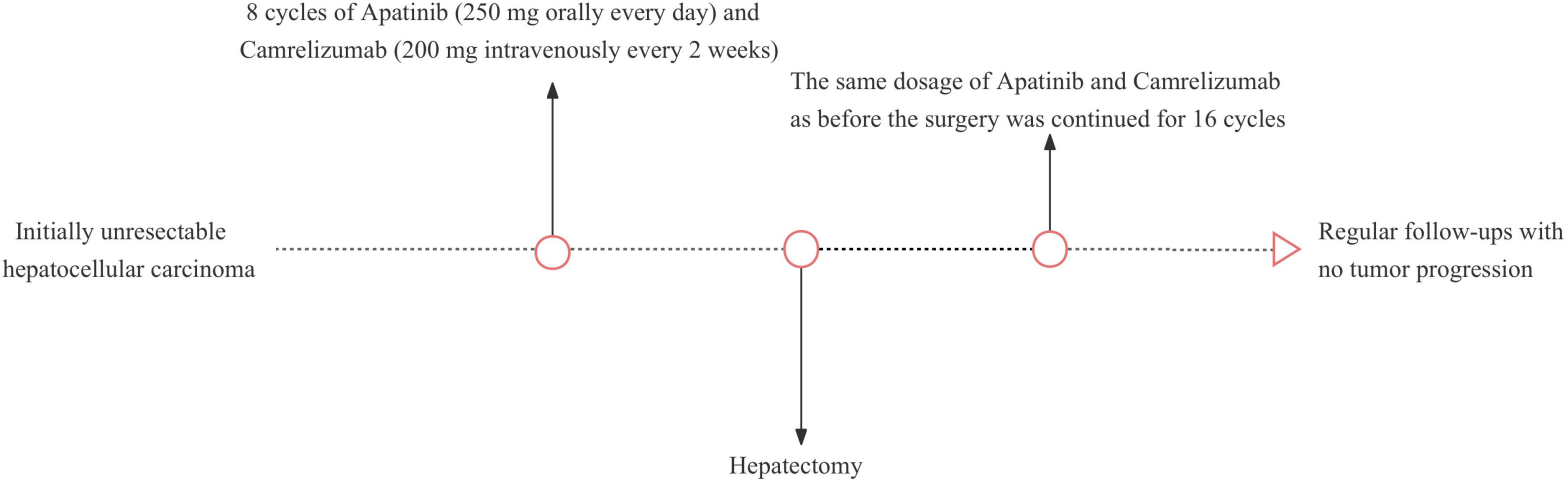
This patient’s treatment history.

An adult patient with right upper abdominal pain was admitted to our hospital. The patient did not undergo antiviral therapy despite having a history of liver cirrhosis caused by the hepatitis B virus for more than 2 years. The albumin concentration was 47.2 (baseline range, 35-50) g/L. The findings of the tumor marker test revealed an AFP level of 21.32 (baseline range, 0-7) ng/ml. A dynamic contrast-enhanced abdominal magnetic resonance imaging (MRI) scan revealed a lesion of approximately 19.1x14.6x10.4cm in size was seen in the right lobe of the liver. Besides, the distal end of the right hepatic vein and the right branch of the portal vein are poorly visualized, which is consistent with invasion by HCC (**Figures 1A, B**). The patient’s HCC was stage IIIA according to the China Liver Cancer staging (CNLC), equivalent to Barcelona Cancer Liver Clinic staging (BCLC) stage C. It is advised that conversion treatment be carried out after the multidisciplinary team (MDT) discussion. As a result, the patient had 8 cycles of conversion therapy with apatinib (250 mg orally every day) and camrelizumab (200 mg intravenously every 2 weeks). The patient also underwent routine hepatoprotective and anti-hepatitis B virus treatments at the same time. We thoroughly examined the patient after the final conversion therapy before the operation. The Eastern Cooperative Oncology Group- Performance Status (ECOG-PS) rating was zero. A liver function test revealed a 38.3 g/L albumin level and a 10.1 (baseline range, 3.42-17.1) umol/l total bilirubin level. Prothrombin time (PT) was 11.8 (baseline range, 11-13) seconds. Thus, the patient’s cirrhosis was categorized as Child-Pugh class A. Following the final conversion treatment, the abdominal contrast-enhanced computed tomography (CT) revealed tumor in the right lobe of the liver shrinks to 8.6x6.9cm (**Figure 1C**) and CT angiography (CTA) showed approximately normal hepatic artery blood flow (**Figure 1D**). No severe adverse effects were noticed during the conversion therapy. These findings led to the conclusion that the HCC had been downstaged to CNLC stage IB, equivalent to BCLC stage A. After the MDT meeting, we decided to operate on this patient and asked that the patient stopped taking medications for four weeks. Preoperative laboratory findings for the patient revealed a level of albumin of 40.2 g/L, a total bilirubin of 8.8 umol/l, and a PT of 11s. The ECOG-PS rating was zero. As a result, the cirrhosis was still given the Child-Pugh class A diagnosis. We further verified by abdominal contrast-enhanced CT that there was no appreciable difference between the preoperative and post-conversion therapy abdominal CTs. This patient also had the indocyanine green retention rate at 15 min (ICG-R15) and the three-dimensional reconstruction of the liver done. ICG-R15 = 7.6%. The liver’s three-dimensional reconstruction (**Figures 1E, F**) revealed a future liver remnant (FLR)/standard liver volume (SLV)=49.6%. The patient’s condition met the requirements for surgery. So, following a preoperative discussion, we conducted a hepatectomy on this patient. The postoperative pathology revealed lymphocyte infiltration, hyperplasia of the surrounding fibrous tissue, and coagulative necrosis of all target lesions (**Figure 1G**). Following surgery, the patient recovered without incident, and no evident problems were discovered. The patient continued to receive the same dose of 16 cycles of apatinib together with camrelizumab 4 weeks after the hepatectomy. By the time of this study, the patient has completed 18 months of follow-up, and no tumor recurrence or metastasis was found in tumor markers and imaging examinations. The patient’s latest follow-up dynamic contrast-enhanced abdominal MRI showed that encapsulated fluid seen at the site of liver surgery with no significant enhancement (**Figures 1H, I**). The treatment history of this patient is detailed in **Figure 2**.

## 3 Discussion

### 3.1 The possibility of apatinib combined with camrelizumab as a conversion therapy

Recently, TKIs and ICIs in patients with unresectable HCC have demonstrated a specific effectiveness profile. It has been reported that systemic therapy, particularly anti-angiogenic drugs combined with immunotherapy, can achieve an objective response rate (ORR) of about 30% and have a median survival time of up to 20 months for patients with advanced or irresectable HCC (15–17). The combination of apatinib and camrelizumab also showed encouraging anticancer activity and a tolerable safety profile in advanced HCC in a phase I study that was begun in October 2016 (18). The combination of apatinib and camrelizumab is effective for the following reasons.

Highly selective tyrosine kinase inhibitor apatinib and program-death receptor 1 (PD-1) inhibitor camrelizumab both work by inhibiting vascular endothelial growth factor 2 (VEGF-2) (19). What’s more, by restoring normalcy to the tumor blood vessels, TKIs are intended to change the hypoxic and immunosuppressive tumor microenvironment (TME) (18). Interferon Gamma (IFN g) + Type 1 T helper (Th1) cells are the predominant populations associated with tumor vascular normalization, and ICIs may increase tumor vascular normalization and activate these cells (20). “Vascular normalization” has the potential to improve therapeutic medication delivery and, more importantly, to reverse immunosuppressive TME by promoting impacted T-cell infiltration into TME, maturation of antigen-presenting cells, and a decrease in immunosuppressive factors (20, 21). TKIs, therefore, improve the effectiveness of ICIs by normalizing the vascular system.

### 3.2 Methods for evaluating the feasibility of surgery

Morphological and functional examinations were undertaken in order to estimate the therapeutic effect before surgery which consists of the overall assessment, the tumor response to conversion therapy, and major side events. First, the ECOG-PS score can be used to evaluate the whole assessment. The patient in our case complied with the following standards: ECOG-PS rating was zero. Second, the modified response evaluation criteria in solid tumors (mRECIST) standard was created to more accurately assess the response of liver lesions because it has advantages in determining the level of pathological response (22, 23). Therefore, in our report, we evaluated the tumor response to therapy using mRECIST criteria. The patient’s tumor target lesion(s), as seen in the abdomen dynamic contrast-enhanced MRI, was lessened. The patient was determined to have a partial response (PR) based on the mRECIST criteria assessment. Last but not least, potential adverse events were assessed primarily based on the patient’s primary complaint, in addition to an electrocardiogram, chest X-ray film, liver and renal function test, cardiac enzymes, regular blood test, standard coagulation test, and other biochemical indicators (24). No severe adverse effects were noticed during the conversion therapy except palmar-plantar erythrodysesthesia and hypertension (Grade 2). In summary, these are the reasons why we consider apatinib in combination with camrelizumab to be effective.

### 3.3 Timing of surgery

Giving patients the chance for radical treatment is the significance of conversion therapy. Our opinion is that through postoperative pathological analysis, surgical resection can not only eradicate any residual tumor cells but also direct adjuvant therapy (25). Based on this, we carefully monitored how the patient’s condition changed before performing drastic surgery on him. Active antiviral therapy and hepatoprotective therapy were provided to the patient during the conversion therapy to meet the surgical requirements (26). As a result, the question of when to undergo surgical treatment has emerged for us to ponder.

Few studies have highlighted the best timing to halt medication therapy before surgery for systemic treatments (27). To assure the safety of a hepatectomy, it is typically required to cease using bevacizumab for 4-6 weeks before surgery based on the experience of treating colorectal liver metastases. According to certain literature findings, HCC conversion surgery should be carried out within four weeks following the conclusion of the previous medication cycle (28). Following a thorough examination of the literature, we discovered that PD-1 inhibitors and small-molecule targeted medications should be discontinued at least two weeks before surgery (25). Therefore, we mandated that the patient stop taking their medications four weeks before surgery. In addition, it is well known that a safe hepatectomy should be performed in patients with normal liver function (Child-Pugh A, ICG-R15 retention rate <10%) and that in patients with chronic liver disease or liver parenchymal damage (including liver cirrhosis, severely fatty liver, and chemotherapy-related liver damage), the sufficient FLR/SLV should be >40% (29). The patient’s cirrhosis was determined to be Child-Pugh class A, and his ICG-R15 was 7.6% before surgery. The three-dimensional reconstruction of the liver can be carried out with the use of various digital imaging tools, which is useful for precisely quantifying the remaining functional liver volume (30). After a comprehensive evaluation, we successfully operated on this patient following preoperative consultations.

### 3.4 Maintenance of postoperative antitumor therapy

It is advised that postoperative adjuvant therapy after R0 resection last for longer than 6 months, with vigilant monitoring occurring every 2-3 months (31). The pharmaceutical dose should be lowered or stopped if severe adverse events or treatment intolerance develop. If there is no recurrence or metastasis in two consecutive imaging tests and tumor markers stay normal, drug discontinuation may be considered (25). As a result, the patient underwent additional 16 cycles of apatinib and camrelizumab following the surgery, during which time he was routinely checked at a nearby hospital every three months. The medication was stopped because there was no evidence of metastasis or recurrence.

## 4 Conclusion

According to the outcomes of our case, the combination of apatinib and camrelizumab may be a successful conversion therapy for HCC that was initially unresectable. Subsequent surgery after conversion therapy with apatinib and camrelizumab may provide patients with long-term oncological benefits. However, subject to sample size, this finding requires more prospective clinical trials to provide higher-level evidence.

## 5 Patient perspective

The doctors and nurses in the hospital are kind to patients, communicate with patients fully, protect patients’ privacy, give proper examinations, and perform proper procedures.

## Data availability statement

The original contributions presented in the study are included in the article/supplementary material. Further inquiries can be directed to the corresponding author.

## Ethics statement

This study was approved by the Medical Ethics Committee of the First Affiliated Hospital of Shandong First Medical University (Shandong Provincial Qianfoshan Hospital) [Number: YXLL-KY-2022(064)]. Written informed consent was obtained from the participant/patient(s) for the publication of this case report.

## Author contributions

X-LL: Writing – original draft. HT: Supervision, Writing – review & editing. X-ZL: Writing – original draft. Y-FC: Writing – original draft. FL: Writing – original draft.
